# Physiology and Metabolism Alterations in Flavonoid Accumulation During Buckwheat (*Fagopyrum esculentum* Moench.) Sprouting

**DOI:** 10.3390/plants13233342

**Published:** 2024-11-28

**Authors:** Meixia Hu, Jia Yang, Jing Zhang, Weiming Fang, Yongqi Yin

**Affiliations:** 1 College of Food Science and Engineering, Yangzhou University, Yangzhou 225009, China; mz120222068@stu.yzu.edu.cn (M.H.); mz120222090@stu.yzu.edu.cn (J.Z.); wmfang@stu.yzu.edu.cn (W.F.); 2 Yangzhou Center for Food and Drug Control, Yangzhou 225000, China; jiajia82112001@163.com

**Keywords:** buckwheat, germination, flavonoids, antioxidant capacity, gene expression

## Abstract

In this research, we investigated the physiological modifications, flavonoid metabolism, and antioxidant systems of two buckwheat (*Fagopyrum esculentum* Moench.) cultivars, Pintian and Suqiao, during germination. The results demonstrated an initial increase followed by a subsequent decline in the flavonoid content of the buckwheat sprouts throughout germination. On the third day of germination, the highest flavonoid concentrations were observed, with the Pintian and Suqiao varieties reaching 996.75 and 833.98 μg/g fresh weight, respectively. Both the activity and relative gene expression level of the flavonoid metabolizing enzyme showed a significant rise in 3-day-old buckwheat sprouts, which was strongly correlated with the flavonoid content. The correlation analysis revealed that the buckwheat sprouts accumulated flavonoids by enhancing the activities and gene expression levels of flavonoid synthases. The antioxidant capacity and the activities and gene expression profiles of the antioxidant enzymes in both buckwheat cultivars notably increased after three days of germination. The correlation analysis indicated a significant positive link between antioxidant capacity and the activity and gene expression levels of the antioxidant enzymes, flavonoid content, and total phenol content. This research demonstrated that germination treatment can significantly boost the accumulation of flavonoids and total phenols, thereby enhancing the antioxidant properties of buckwheat sprouts, despite variations among different buckwheat varieties.

## 1. Introduction

Buckwheat (*Fagopyrum esculentum* Moench.)is cultivated and eaten worldwide [1]. Recently, with increasing awareness of healthy eating, buckwheat has gained attention for its nutrients and bioactive substances. Buckwheat is a pseudocereal that, unlike grains with nutrients (carbohydrates, proteins, and vitamins), is high in protein, containing between 7 and 21% [2]. Buckwheat is also a significant source of essential minerals. It contains high levels of copper, manganese, and zinc, which are crucial for maintaining various bodily functions [3]. Meanwhile, buckwheat, as a cereal crop abundant in bioactive compounds, features a remarkable chemical composition where total flavonoids hold a significant position [4]. Flavonoids, a class of polyphenolic secondary metabolites widely found in the plant kingdom, not only bestow buckwheat with beneficial properties such as antioxidant [5], anti-inflammatory [6], and blood pressure-lowering [7] properties. Meanwhile, clinical studies have shown that flavonoids extracted from buckwheat mainly inhibit the growth of human acute myeloid leukemia HL-60 cells by increasing flow cytometry annexin binding capacity and activating caspase 3 protein, thereby achieving anti-cancer properties [8]. In addition, flavonoids have become a focal point for the scientific community to conduct in-depth research into their functional mechanisms and potential applications. Therefore, the synthesis of flavonoids in plants has received more and more attention. However, the content of flavonoids is influenced by various environmental factors.

As an inherent bioprocessing methodology, germination significantly enhances the biological activity and functional attributes of plants [9]. Post-germination, the macromolecules of starch and protein within the seeds are decomposed into simpler nutrients, thereby facilitating easier absorption and utilization by the human body [10]. Concurrently, during the germination process, plants synthesize an array of bioactive constituents, encompassing phenolic acids [11] and polyphenols [12]. Liu et al. [13] concurrently germinated 17 types of seeds and observed a substantial accumulation of phenolic acids and flavonoids in the resulting sprouts. Ji et al. [14] established a significant relationship between the accumulation of flavonoids and the primary enzymes responsible for their biosynthesis. The germination of seeds initiates a series of intricate physiological and biochemical transformations, with the activation of the antioxidant enzyme system being of particular importance. Peng et al. [15] found that the contents of total phenols, total flavonoids, and antioxidant capacity in buckwheat sprouts were higher than those in buckwheat seeds. Zhang et al. [16] studied the germination process of seeds and found that flavonoid content, total phenol, and the activity of four antioxidant enzymes were enhanced. Research has confirmed that there are significant differences in the composition and content of flavonoids among various buckwheat cultivars [17,18,19,20] and different tissues [21,22] of buckwheat sprouts. Generally, the flavonoid content in bitter buckwheat sprouts is higher compared to that in common buckwheat, particularly in the cotyledons, where flavonoid levels surpass those in other tissue regions. In recent years, an increasing number of studies have focused on controlling germination conditions to enhance flavonoid accumulation in buckwheat sprouts. Appropriate light intensity [23,24], light sources [25,26], ultraviolet irradiation [14,27,28], laser treatment [10], exogenous compounds [29,30,31,32], and microwave treatment [33] have all been shown to significantly increase flavonoid content in buckwheat sprouts. Consequently, the antioxidant capacity of these flavonoid-rich buckwheat sprouts has also been markedly enhanced, establishing a foundational basis for developing nutritional foods or dietary supplements derived from buckwheat sprouts [34]. Furthermore, the enzymes involved in flavonoid metabolism are crucial for the synthesis and accumulation of flavonoids during the process of plant germination. Plant flavonoids are primarily synthesized via the phenylpropanoid metabolic pathway. The enzyme activities of phenylalanine ammonia lyase (PAL), cinnamic acid 4-hydroxylase (C4H), 4-coumarate coenzyme A ligase (4CL), and chalcone isomerase (CHI) within the flavonoid biosynthetic pathway are intricately linked to the gene expression of these respective enzymes, as well as to the synthesis and accumulation of flavonoids [35]. Ren et al. [36] demonstrated a significant positive linear relationship between PAL activity and the accumulation of flavonoids in both common and bitter varieties of buckwheat (*p* < 0.01). Chen et al. [37] observed an increase in the concentration of phenolic compounds in sprouting buckwheat, a phenomenon that was modulated by the enzymatic activities of PAL and CHI. Similarly, Li et al. [38] reported that during the germination of barley, there was an elevation in the activities of PAL, C4H, and 4CL, which corresponded with the accumulation of phenolic compounds. In addition, Ma et al. [39] investigated the impact of microwave irradiation on the expression levels of phenylalanine ammonia-lyase (PAL), chalcone isomerase (CHI), and flavanol synthase (FLS), as well as the concentration of flavonoid substances in buckwheat sprouts. Furthermore, Rao et al. [40] utilized quantitative proteomics to reveal that mildly acidic electrolyzed water induces the accumulation of flavonoid compounds by increasing the abundance of enzyme proteins in the phenylpropanoid biosynthetic pathway within buckwheat sprouts. These studies, however, did not examine the enzymatic activity and comparative gene expression profiles of flavonoid biosynthetic enzymes across the complete metabolic pathway of phenylpropanoids during the germination process of genetically engineered buckwheat sprouts.

This research aimed to explore the regulatory mechanisms of antioxidant activity and flavonoid accumulation in two cultivars (Pintian and Suqiao) during germination. Initially, the physiological and biochemical dynamics throughout the growth of buckwheat sprouts were examined. Subsequently, the alterations in the antioxidant system during germination were disclosed. Finally, the molecular mechanisms behind flavonoid accumulation in buckwheat were elucidated by assessing the functional activity and transcriptional levels of flavonoid synthases throughout germination. The significance of this study lies in providing a theoretical foundation for comprehending the mechanism of flavonoid accumulation during buckwheat germination and offering technical support for the production of functional foods enriched with flavonoids.

## 2. Results

### 2.1. Changes in Buckwheat Sprouts in Biochemical and Physiological Indexes

**Figure 1:**
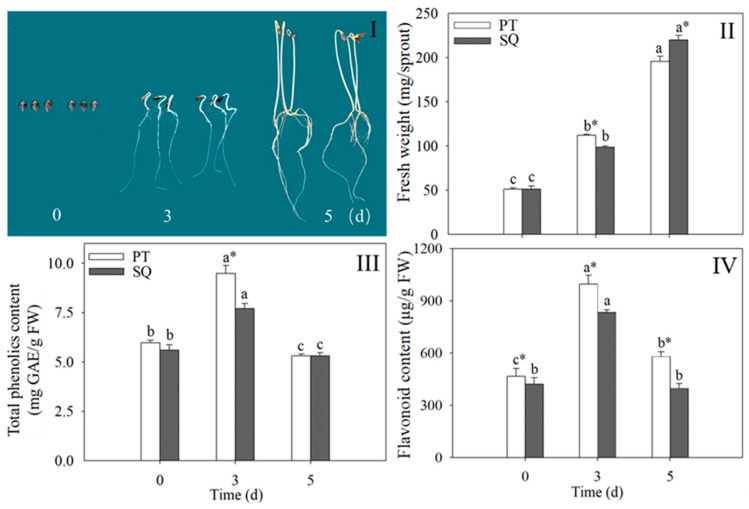
Changes in growth morphology (**I**), fresh weight (**II**), total phenolic content (**III**), and flavonoid content (**IV**) in buckwheat sprouts. Distinct lowercase letters signify statistically significant differences in germination times for the same buckwheat cultivar (*p* < 0.05). Asterisks (*) denote significant differences among buckwheat cultivars at identical germination times (*p* < 0.05). PT: Pintian. SQ: Suqiao.

The biochemical and physiological transformations of buckwheat sprouts during the germination phase are illustrated in Figure 1. The sprout growth is visibly apparent (Figure 1I). The fresh weight of the sprouts also increases during the growth phase (Figure 1II). Throughout germination, the overall phenolic and flavonoid concentrations present in buckwheat sprouts initially rise and then fall, with peak values occurring after 3 days of germination (Figure 1III,IV). Compared to ungerminated buckwheat (0 d), the flavonoid contents of the Pintian sprouts and Suqiao sprouts increased by 53.2% and 49.3%, respectively. Moreover, a notable disparity in the total phenolic and flavonoid content was identified between the Pintian and Suqiao sprouts following 3 days of germination (*p* < 0.05). At this point, the total phenolic and flavonoid concentrations in the Pintian sprouts were found to be 1.3 and 1.2 times greater, respectively, than those in the Suqiao sprouts. These findings suggest that while the overall trend in total phenolic and flavonoid contents during germination is similar across different buckwheat cultivars, the extent of change varies.

### 2.2. Changes in Antioxidant Capacity in Buckwheat Sprouts

**Figure 2:**
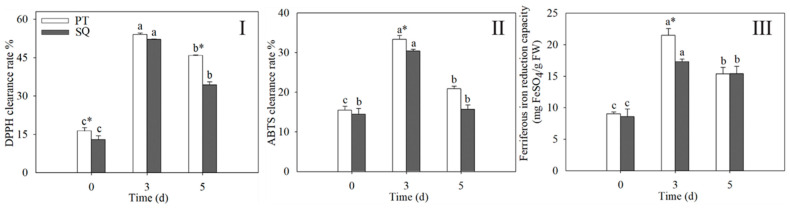
Changes in the capacities of DPPH (**I**), ABTS (**II**), and FRAP (**III**) in buckwheat sprouts are shown. Distinct lowercase letters signify statistically significant differences in germination times for the same buckwheat cultivar (*p* < 0.05). Asterisks (*) denote significant differences among buckwheat cultivars at identical germination times (*p* < 0.05). PT: Pintian. SQ: Suqiao.

Throughout the germination phase, the antioxidant capacity initially increased, peaking at three days, and subsequently declined in the buckwheat sprouts (Figure 2). On the third day following the initiation of germination, the antioxidant activity exhibited a statistically significant increase in comparison with both 0 d and 5 d (*p* < 0.05). Following a three-day period of germination, the antioxidant capacities, as measured by ABTS and FRAP, were notably greater in the Pintian sprouts compared with the Suqiao sprouts—exceeding them by factors of 1.1 and 1.2, respectively. However, no notable variation in DPPH activity was detected between the Pintian and Suqiao sprouts after three days (*p* > 0.05). This suggests that the antioxidant capacities among different varieties of buckwheat vary throughout their growth stages.

### 2.3. Changes in the Activity and Gene Expression Levels of the Flavonoid Synthase Enzyme in Buckwheat Sprouts

**Figure 3:**
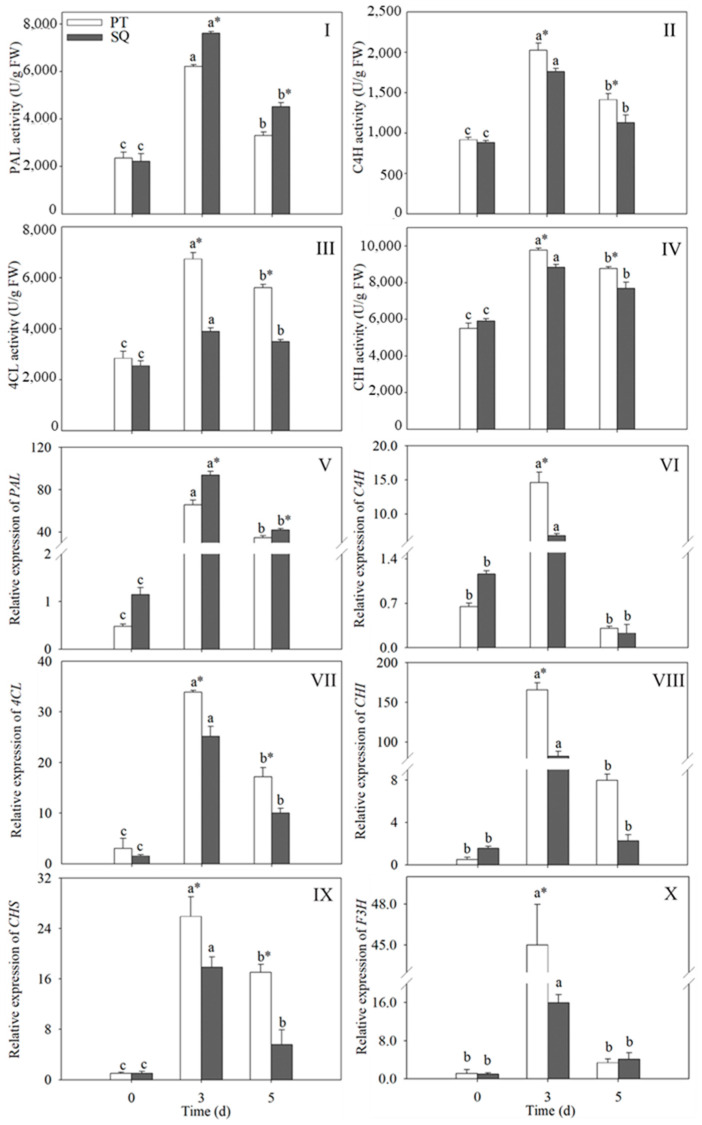
Changes in the activities of PAL (**I**), C4H (**II**), 4CL (**III**), and CHI (**IV**) and the expression levels of *PAL* (**V**), *C4H* (**VI**), *4CL* (**VII**), *CHI* (**VIII**), *CHS* (**IX**), and *F3H* (**X**) in buckwheat sprouts. Distinct lowercase letters signify statistically significant differences in germination times for the same buckwheat cultivar (*p* < 0.05). Asterisks (*) denote significant differences among buckwheat cultivars at identical germination times (*p* < 0.05). PT: Pintian. SQ: Suqiao.

Figure 3 depicts the changes in both the activity and gene expression levels of the flavonoid synthetase. The observed trend exhibits a preliminary rise succeeded by a decline, with the peak value occurring at 3 days post-germination. Compared to ungerminated buckwheat (0 d), a notable increase (*p* < 0.05) was observed in both the activity and comparative gene expression of flavonoid-metabolizing enzymes at 3 days of germination. However, different buckwheat cultivars displayed variable enzymatic activities and gene expression profiles at the identical germination phase. Specifically, the activity of PAL in the Suqiao sprouts was observed to be 1.2 times greater than that in the Pintian sprouts after 3 days of germination (Figure 3I). Conversely, the Pintian sprouts exhibited elevated activities of C4H, 4CL, and CHI, with increases of 1.3, 1.6, and 1.2 times, respectively, when compared to the Suqiao sprouts (Figure 3II–IV). The enzymatic activity and associated gene expression levels related to flavonoid metabolism in both cultivars exhibited statistically significant differences on the third day of germination (*p* < 0.05). Additionally, the findings in Figure 3I were corroborated by Figure 3V, demonstrating a marked increase in PAL expression levels in the Suqiao sprouts compared to the Pintian sprouts at 3 days of germination. However, at 3 days of germination, the corresponding gene expression levels of the flavonoid-metabolizing enzymes (C4H, 4CL, CHI, Chalcone synthase (CHS) and Flavanone 3-hydroxylase (F3H)) in the Pintian sprouts were significantly increased compared to the Suqiao sprouts (*p* < 0.05) (Figure 3VI–X).

### 2.4. Changes in the Activities and Gene Expression Levels of Antioxidant Enzymes in Buckwheat Sprouts

**Figure 4:**
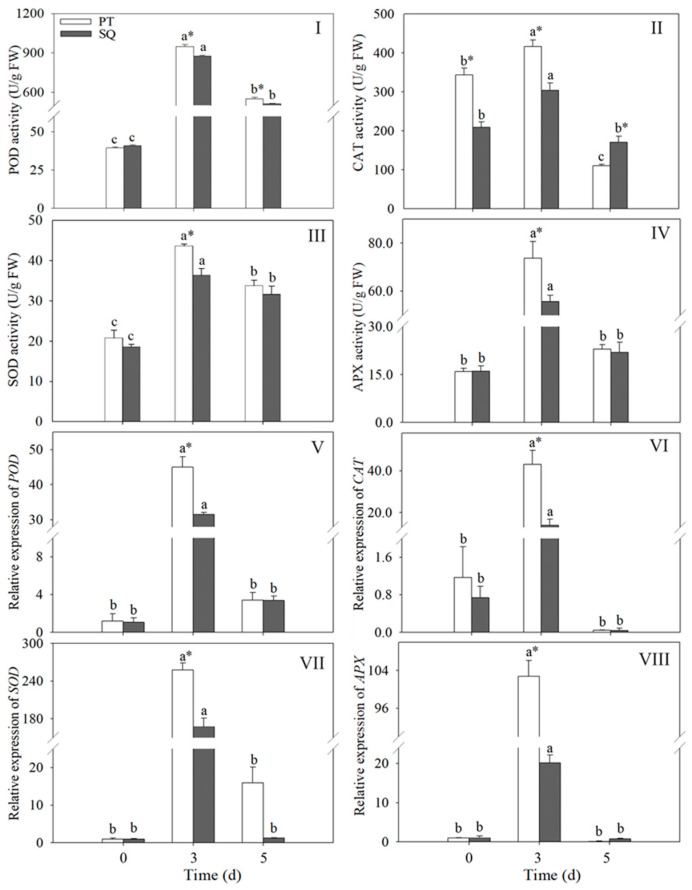
Changes in the activity of POD (**I**), CAT (**II**), SOD (**III**), and APX (**IV**) and the expression levels of *POD* (**V**), *CAT* (**VI**), *SOD* (**VII**), and *APX* (**VIII**) in buckwheat sprouts. Distinct lowercase letters signify statistically significant differences in germination times for the same buckwheat cultivar (*p* < 0.05). Asterisks (*) denote significant differences among buckwheat cultivars at identical germination times (*p* < 0.05). PT: Pintian. SQ: Suqiao.

Throughout the germination phase, the activities and gene expression patterns of the antioxidant enzymes in the buckwheat sprouts exhibited an initial increase followed by a subsequent decline, with the highest levels observed at 3 days of germination. Compared to 0 d and 5 d, the activities and gene expression levels of the antioxidant enzymes in the buckwheat sprouts exhibited a significant increase following three days of germination (*p* < 0.05). Furthermore, following a three-day germination period, there were notable differences in both the activity and gene expression levels of the antioxidant enzymes between the Pintian sprouts and Suqiao sprouts (*p* < 0.05). Specifically, at three days post-germination, the activities of POD, CAT, SOD, and APX in the Pintian sprouts were observed to be 1.1, 1.4, 1.2, and 1.3 times greater than those in the Suqiao sprouts, respectively (Figure 4I–IV). Concurrently, the expression levels of *POD*, *CAT*, *SOD*, and *APX* in the Pintian sprouts were 1.1, 3.1, 1.5, and 5.1 times those of the Suqiao sprouts, respectively, after 3 days of germination (Figure 4V–VIII).

### 2.5. The Pearson Correlation Analysis of Flavonoids with Various Indexes

**Figure 5:**
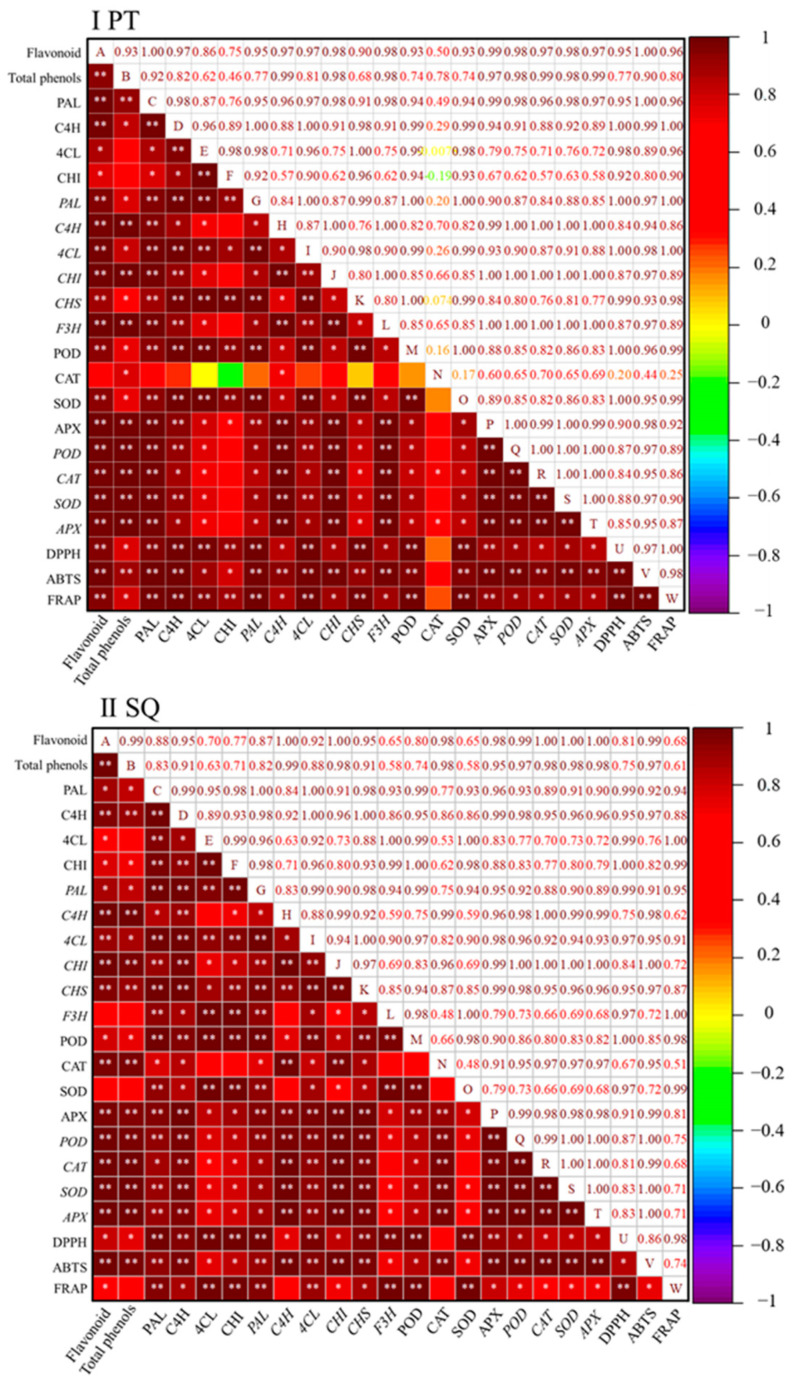
The examination of the correlation analysis of flavonoids with various indexes in Pintian sprouts and Suqiao sprouts is represented by (**I**) and (**II**), respectively. Negative correlations are indicated by shades of purple, blue, and green, whereas positive correlations are represented by shades of red. The correlation coefficients can vary from −1 to 1. Asterisks (*) and (**) denote that the correlation coefficient is statistically significant at *p*-value thresholds of 0.05 and 0.01, respectively. PT: Pintian; SQ: Suqiao.

The Pearson correlation analysis examining the relationship between flavonoid content and the activities and gene expression levels of antioxidant enzymes, as well as the flavonoid-metabolizing enzyme, during the germination of buckwheat is presented (Figure 5). The aim of this study was to elucidate the correlation between flavonoid levels in sprouts and various biological markers. In both the Pintian and Suqiao sprouts, the flavonoid content demonstrated a notable positive correlation with the activities of PAL, C4H, 4CL, and CHI, and the expression levels of *PAL*, *C4H*, *4CL*, *CHI*, and *CHS*. Additionally, in the Pintian sprouts, a significant positive correlation was observed between the flavonoid content and *F3H* (*p* < 0.01), but no significant correlation was found between the flavonoid content and *F3H* in the Suqiao sprouts. The flavonoid content in the Pintian sprouts and Suqiao sprouts exhibited a substantial positive correlation with the activities of POD and APX, as well as the expression levels of *POD*, *CAT*, *SOD*, and *APX*. In the interim, a significant positive correlation was observed between the flavonoid content in the Pintian and Suqiao sprouts and their respective capacities for DPPH, ABTS, and FRAP. In the Pintian sprouts, the flavonoid concentration exhibited a notable positive association with SOD activity (*p* < 0.01) but not with CAT activity. Conversely, in the Suqiao sprouts, the flavonoid concentration exhibited a notable positive association with CAT activity (*p* < 0.01) but showed no significant correlation with SOD activity.

### 2.6. Antioxidant Changes and Regulatory Mechanism of Flavonoid Metabolism in Buckwheat During Germination

**Figure 6:**
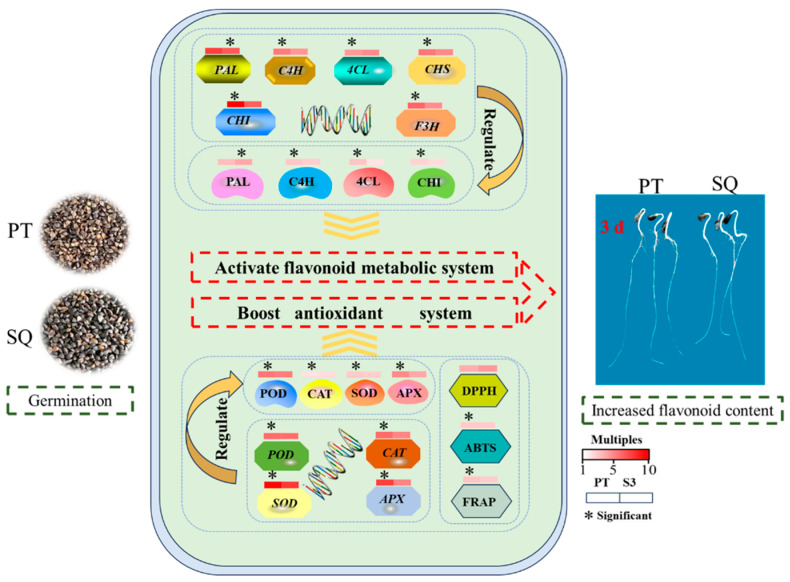
The mechanism diagram of germination that regulates the antioxidant system and the flavonoid metabolism system of buckwheat sprouts.

Figure 6 depicts the predicted molecular process of the flavonoid production in the two buckwheat cultivars after three days of germination. At this moment, the flavonoid concentration in both cultivars had peaked. The higher flavonoid content in these cultivars is due to flavonoid production being regulated by flavonoid-metabolizing enzyme activity and gene expression levels. However, after three days of germination, the flavonoid content differed between the two cultivars, owing mostly to changes in C4H, 4CL, and CHI activity, as well as expression levels of *C4H*, *4CL*, *CHI*, *CHS*, and *F3H.* Furthermore, the antioxidant system in both germinates was active after three days, as evidenced by the antioxidant enzyme activity and gene expression. Interestingly, the antioxidant content of the Pintian sprouts topped that of the Suqiao sprouts.

## 3. Discussion

Flavonoids represent the most prevalent natural secondary metabolites of polyphenols, generated through the phenylpropanoid pathway in plants during their expansion and progression [41]. Beyond facilitating the growth and development of plants, their antioxidant properties significantly contribute to human health. Flavonoids are ubiquitously present in most plant tissues [42]. Notably, there is a substantial increase in flavonoid content during the germination of seeds [43]. During the germination process, different sprout species exhibit varying levels of flavonoids. For instance, among four types of sprouts, sunflower sprouts possess the highest flavonoid concentration, having a concentration 1.2, 1.3, and 3.2 times greater than that found in broccoli sprouts, radish sprouts, and mung bean sprouts, respectively [44]. Research has confirmed that the predominant flavonoids present in buckwheat sprouts include orientin, isoorientin, vitexin, isovitexin, rutin, quercetrin, and quercetin, with varying responses to external treatments among different flavonoid monomers [17,45]. For instance, blue light treatment resulted in a notable increase in the levels of C-glycosyl flavonoids (orientin, vitexin and its isomers, and rutin and its isomers) [25]. The study by Ren et al. [36] indicated that throughout the germination phase, the concentrations of total flavonoids and rutin in both varieties of buckwheat exhibited a progressive increase, reaching their maximum levels on the 7th and 9th days of germination, respectively. In contrast, the quercetin content in bitter buckwheat begins to decline from the 5th day and becomes undetectable by the 7th day. This highlights the considerable influence of germination duration on the biosynthesis of total flavonoids and flavonoid monomers across various buckwheat cultivars. Although our current study did not assess the changes in monomeric flavonoids during the germination of the two buckwheat varieties, our research discovered that as the germination time increased, the flavonoid content of the buckwheat initially rose and then declined, peaking at three days of germination. However, the flavonoid content showed a significant difference (*p* < 0.05), with the Pintian sprouts containing 996.75 μg/g fresh weight of flavonoids, which was 1.2 times higher than that of the Suqiao sprouts. Lv et al. [46] observed that most broccoli sprouts obtained peak flavonoid content at 3 days old. The results of this investigation are consistent with the existing literature, demonstrating an initial increase in flavonoid content during the germination process, followed by a subsequent decrease. Furthermore, these fluctuations were observed to differ among the various sprout cultivars. To further investigate the variations in flavonoid biosynthesis during germination in these two buckwheat varieties, it is essential to analyze the trends in monomeric flavonoid changes in further research. Additionally, while earlier research has demonstrated an absence of a significant relationship between the leaf length of common buckwheat sprouts and their rutin content, a notable positive correlation has been identified between leaf length and rutin content in bitter buckwheat sprouts [21]. However, in our study, the physiological states of the two buckwheat seedlings (sprout length and fresh weight) observed a notably substantial positive correlation with total flavonoid content. Previous research has demonstrated that the ethanol extract of buckwheat sprouts exhibits significant reducing power, as well as notable free radical scavenging and superoxide anion scavenging activities, effectively inhibiting the production of peroxides within human liver cancer HepG2 cells and eliminating intracellular superoxide anions [19]. Research indicates that treatments utilizing blue light [25], a combination of microwave and l-phenylalanine [15], sucrose [30], and ultraviolet light [14] have led to buckwheat sprouts demonstrating not only the highest levels of total flavonoids and total phenolic compounds but also the most significant antioxidant activity. Lee et al. [47] demonstrated a significant correlation between the antioxidant capacity of buckwheat sprouts and the concentration of flavonoids present.

The research also examined fluctuations in the antioxidant potential of buckwheat throughout its developmental stages, specifically assessing DPPH, ABTS, and FRAP capacities. The results indicated that as the germination time increased, the capacities of DPPH, ABTS, and FRAP in the buckwheat initially rose before diminishing, with the highest levels recorded at three days of germination. Shabbir et al. [48] also demonstrated that the antioxidant capacity of germinated black bean sprouts significantly improved, as evidenced by DPPH, ABTS, and FRAP assays. The upward trend in antioxidant capacity in the buckwheat sprouts corresponded with increased activity of antioxidant enzymes and demonstrated a notable positive correlation with the content of flavonoids. Similarly, Lee et al. [47] demonstrated a significant correlation between the antioxidant capacity of buckwheat sprouts and the concentration of flavonoids present.

The fundamental metabolic mechanism involved in flavonoid production in buckwheat is facilitated by four enzymes: PAL, C4H, 4CL, and CHI. The enzyme-catalyzed conversion of phenylalanine by PAL, C4H, and 4CL leads to the formation of 4-coumaryl CoA. Under conditions of salt stress, the content of flavonoids is increased through the regulation of both the phenylpropanoid and flavonoid biosynthetic pathways, as well as the enzymatic activities related to these pathways [49]. Melatonin is integral to mediating flavonoid synthesis genes, significantly promoting flavonoid accumulation [50]. The principal enzymes implicated in the biosynthetic metabolism of flavonoids in buckwheat sprouts subjected to blue light and ultraviolet treatment exhibited a notable correlation with the overall flavonoid content [14]. The administration of sucrose has been shown to promote the accumulation of flavonoids in buckwheat sprouts by elevating the enzymatic activity of tyrosinase and phenylalanine ammonia-lyase [30]. The study observed that the activities of PAL, C4H, 4CL, and CHI initially increased and then decreased over the germination period. This pattern was consistent with the observation that the concentration of flavonoids diminished with increased germination duration. Notably, while the flavonoid content in the Pintian sprouts exceeded that of the Suqiao sprouts, the latter exhibited higher PAL activity compared to the Pintian sprouts. The phenylpropane metabolism pathway was catalyzed by a variety of enzymes. While the PAL activity in the Pintian sprouts was lower, the activities of C4H, 4CL, and CHI were higher compared to the Suqiao sprouts. These findings suggest that buckwheat germination promotes the accumulation of flavonoids by increasing the activities of PAL, C4H, 4CL, and CHI. The content of flavonoids exhibited a notable positive correlation with the activities of the four flavonoid synthases (PAL, C4H, 4CL, and CHI). Prior research has additionally shown a positive association between flavonoids and the effects of PAL, C4H, 4CL, and CHI [51]. Furthermore, it was proved that treatment with microwave radiation significantly enhances the transcription of multiple genes associated with flavonoid biosynthetic enzymes in bitter buckwheat sprouts, consequently facilitating the accumulation of flavonoids [39]. An analysis of gene expression at the molecular level may yield a more comprehensive understanding of the mechanisms that govern flavonoid accumulation during the germination process of buckwheat. During the germination process, the quantification of *PAL*, *C4H*, *4CL*, *CHI*, *CHS*, and *F3H* exhibited an initial increase, peaking on the third day, after which a decline was observed. Compared to non-germinated buckwheat (0 d), the quantification of flavonoid-metabolizing enzyme genes in the buckwheat sprouts had a notable upregulation on the third day of germination. However, the relative expression levels varied across the different buckwheat cultivars. For instance, on the third day of germination, the Suqiao sprouts exhibited higher relative expression levels of *PAL* than the Pintian sprouts, which correlated with PAL activity. The same expression levels of *PAL*, *C4H*, *4CL*, *CHI*, *CHS*, and *F3H* for flavonoids in buckwheat have been also recognized in prior research studies [52].

In the in-depth study of plant physiology and its adaptive mechanisms, a prominent area of focus is on the “dynamic regulation of the antioxidant enzyme system during the germination process”. This process not only illustrates the complex reactions of living organisms to environmental fluctuations in their natural surroundings but also emphasizes the essential function of antioxidant enzymes in facilitating the transition of seeds from dormancy to active growth. With the germination of seeds, a series of complex physiological and biochemical changes are triggered, among which the activation of the antioxidant enzyme system is particularly crucial. In this investigation, the activities and quantification of antioxidant enzymes were measured throughout the developmental stages of buckwheat. The findings indicated that the gene expression levels of the antioxidant enzymes were consistent with their activities in the buckwheat sprouts. Specifically, the activities and mRNA transcription quantities of CAT, POD, and APX were increased significantly compared to those of seeds (*p* < 0.05) during the third day of germination. This trend mirrors the changes in flavonoid content during buckwheat sprout development. Additionally, the flavonoid content in buckwheat sprouts exhibited a significant positive correlation with the activities and gene expression levels of antioxidant enzymes. Similarly, prior research has shown a positive correlation between flavonoid content and antioxidant activity in vegetable sprouts [53].

## 4. Materials and Methods

### 4.1. Germination Process

Buckwheat seeds (*Fagopyrum esculentum*), the Pintian and Suqiao cultivars, were planted in 2022 and sourced from the Institute of Agricultural Sciences in Taixing City, Jiangsu Province. After the seeds were sterilized and germination was performed. The buckwheat sprouts were systematically sampled at predetermined intervals (0, 3, and 5 days) for subsequent analysis.

### 4.2. Measurement of Principal Physiological and Biochemical Parameters

The fresh weight of 30 buckwheat sprouts with uniform growth was measured. Every 10 buckwheat sprouts were rinsed and wiped dry to remove surface moisture before weighing. The total fresh weight of the 10 buckwheat sprouts was divided by 10 to obtain the average fresh weight of each sprout. The above experiments were repeated three times. Flavonoids were extracted and quantified according to the methodology of Yin et al. [54]. In this experiment, a total of fresh buckwheat sprouts (1.0 g) was initially subjected to grinding using 6 mL of 80% (*v*/*v*) ethanol solution, resulting in a homogenate that exhibited no discernible plant tissue. Subsequently, this homogenate underwent sonication for a duration of 25 min at a controlled temperature of 25 °C to further enhance the extraction process. The homogenate was then centrifuged. From this supernatant, an aliquot of 0.5 milliliters was meticulously withdrawn and subsequently diluted 25-fold with the same 80% (*v*/*v*) ethanol solution. The absorbance of the prepared solution was then measured at a wavelength of 260 nm.

The total phenolic compounds were extracted and quantified following Zhang et al. [55] protocol. The buckwheat sprouts (1.0 g) are ground with 5 mL of 50% (*v*/*v*) methanol, and the supernatant is collected by centrifugation. Next, 1 mL of the supernatant is mixed with 1 mL of 0.2 mM Folin–Ciocalteu reagent and 2 mL of 2% (*w*/*v*) sodium carbonate (Na_2_CO_3_), and then treated in the dark for 2 h. The total phenolic content is quantified by measuring the absorbance at a wavelength of 765 nm.

### 4.3. Assessment of Antioxidant Capacity

Antioxidant capacities were measured for 1,1-diphenyl-2-picrylhydrazyl (DPPH) and 2,2′-azino-bis(3-ethylbenzothiazoline-6-sulfonic acid) (ABTS) using the Alfuraydi et al. [56] method. To begin with, a total of 1.0 g of freshly harvested millet sprouts was subjected to extraction using 5 mL of a 50% (*v*/*v*) methanol solution. Subsequently, 2 mL of DPPH (2,2-diphenyl-1-picrylhydrazyl) solution, prepared at a concentration of 0.2 mM in ethanol, was added to 1 mL of the previously obtained extraction solution. The absorbance levels of the resulting mixture were then measured at a wavelength of 517 nm. For comparison purposes, distilled water was utilized as the control in this experimental setup.

The ABTS cation radical was produced through a chemical reaction involving a 2.45 mM solution of potassium persulfate (K_2_S_2_O_8_) in combination with a 7 mM aqueous solution of ABTS, maintaining a ratio of 1:1. This mixture was allowed to stand for 12 h in a dark environment before its application. The ABTS activity assay was modified, the working solution was diluted 8-fold with absolute ethanol, and then 0.1 mL of the extract was mixed with 4.9 mL of the working solution.

Ferriferous ion reduction capacity (FRAP) was evaluated according to Zhang et al. [57]. A total of 100 µL of the extract was placed into a test tube, followed by an adjustment of the volume to 1 mL using methanol as the solvent. Subsequently, 2.5 mL of phosphate buffer was added, along with 2.5 mL of a 1% potassium ferricyanide solution. The contents of the test tube were then subjected to vortex mixing to ensure thorough mixing of the components. The resulting mixture was allowed to incubate for a duration of 20 min at a controlled temperature of 50 °C within a water bath. After the incubation, 2.5 mL of 10% (*w*/*v*) trichloroacetic acid was introduced to the mixture and centrifuged at a force of 8000*× g* for a period of 20 min. Following centrifugation, 2.5 mL of the resultant supernatant was extracted and combined with 2.5 mL of distilled water, along with 0.5 mL of a 0.1% (*w*/*v*) ferric chloride solution within another test tube. Finally, the absorbance of this solution was measured at a wavelength of 700 nm. It is important to note that a higher absorbance value is indicative of an increased reducing power of the extract being analyzed.

### 4.4. Enzymatic Activity Measurement of Flavonoid Biosynthesis

The activities of phenylalanine ammonia lyase (PAL), cinnamic acid 4-hydroxylase (C4H), and 4-coumarate coenzyme A ligase (4CL) were analyzed as per Yin et al. [58]. After grinding the buckwheat sprouts with Tris-HCl buffer solution, a centrifuge was used to collect the supernatant. For the purpose of this study, one unit of enzyme activity for phenylalanine ammonia-lyase (PAL), cinnamate-4-hydroxylase (C4H), and 4-coumarate-CoA ligase (4CL) was defined as a measurable change of 0.01 per minute in optical density readings at 290 nm, 340 nm, and 333 nm, respectively.

The determination of chalcone isomerase (CHI) activity was evaluated utilizing the methodology outlined by Ji et al. [14]. The supernatant obtained from the prior extraction process was utilized as a source of crude enzyme. Then, the enzyme solution was mixed with the reaction solution and allowed to react for 30 min. The evaluation of chitinase (CHI) activity was conducted by monitoring the changes in absorbance at a wavelength of 381 nm.

### 4.5. Evaluation of Antioxidant Enzyme Activities

Buckwheat sprouts underwent a grinding process utilizing sodium phosphate buffer at a pH of 7.0, with a concentration of 50 mM. Following this initial preparation, the resultant homogenate was subjected to centrifugation at 12,000× *g* for a duration of 15 min at 4 °C. This procedure effectively facilitated the separation of the supernatant from the solid residue.

The activities of catalase (CAT) and peroxidase (POD) were evaluated following the procedure of Zhang et al. [59]. A single unit of activity for both CAT (catalase) and POD (peroxidase) is meticulously defined as a variation or change of 0.01 per minute when measured at optical densities of 240 nm and 470 nm, respectively.

The activities of superoxide dismutase (SOD) and ascorbate peroxidase (APX) were determined using the method of Bin et al. [60]. A specific unit for measuring the activity of superoxide dismutase (SOD) and ascorbate peroxidase (APX) was established, which is characterized by a variation of 0.01 per minute at optical densities of 560 nm and 290 nm, respectively.

### 4.6. RNA Extraction and Quantitative Real-Time PCR Analysis

Buckwheat sprouts were treated with the E.A.N.A.TM Plant RNA Kit (R6827-01, Omega, Norcross, GA, USA) to extract total RNA. After being extracted, the RNA was reverse transcribed using the PrimeScript^TM^ RT Master Mix Kit (RR036A, Takara Bio, Kusatsu, Japan) into complementary cDNA. Each cDNA sample was evaluated in triplicate for quantitative analysis using the Light Cycler 480 II real-time PCR equipment (Roche, Basel, Switzerland) and SYBR Premix Ex-Taq^TM^ II (RR820S, Takara Bio, Kusatsu, Japan). Supplementary Table S1 provides specific information on the oligonucleotide primers used in the quantitative reverse transcription (qRT-PCR). The 2^−ΔΔCt^ comparative approach was conducted to ascertain the relative expression levels of genes.

### 4.7. Statistical Analysis

All experiments were performed in triplicate, and the results are presented as the mean ± standard deviation. The DPS data processing system software (2024) was used for statistical analysis. Statistical analysis employed one-way ANOVA and Tukey’s multiple comparisons test, with significance thresholds set at *p* < 0.05 and *p* < 0.01.

## 5. Conclusions

In summary, two buckwheat cultivars were used to assess germination and flavonoid accumulation. Additionally, the physiology, flavonoid metabolism, and antioxidant system during buckwheat germination were investigated. The results showed that germination treatment greatly increased flavonoid and total phenol accumulation, as well as the antioxidant capacity of the buckwheat sprouts. Nevertheless, significant differences were detected between the cultivars.

## Data Availability

The data presented in this study are available on request from the corresponding author.

## Supplementary Materials

The following supporting information can be downloaded at: https://www.mdpi.com/article/10.3390/plants13233342/s1, Table S1: The sequences of the primers utilized in the study.
