# Supplementary material for: Physiology and Metabolism Alterations in Flavonoid Accumulation During Buckwheat (Fagopyrum esculentum Moench.) Sprouting
Source: Plants (Basel). 2024 Nov 28;13(23):3342. doi: 10.3390/plants13233342 (PMC11644169; doi:10.3390/plants13233342)
Supplement: Supplementary file 1 [file plants-13-03342-s001.zip › plants-3287259-supplementary.pdf]

Table S1

Primer sequences used in the study.

Primer	Sequences (5'-3')	
Actin	F[1] :TCGTGAGAAGATGACCCAGA	R[2] :CCGAGTCCAGCACAATACCT
PAL	F :TCTCCAGAAGCCGAAACAAG	R :AGCCTTGTTCCTGGATACAT
C4H	F :AACACACTACTCTCAGTTGC	R :ATTGGGTGATCGAGACTCTT
4CL	F :CTCTTTCACGTCCACGGTTT	R :GATGATTTGGTGGATGGTGG
CHS	F :CGTCAAGCGTTTCATGATGT	R :CAAGGCTTGTGTTGACATGG
CHI	F :ACTTTGAGGAATCCGCTGTGAC	R :AGGGCTTCAACATGGTGATCTGTA
F3H	F :CAAGGCTTGTGTTGACATGG	R :GACAGTGATCCAGGTCTTGC
CAT	F :GAGTTTGGTTCCCTTGCTT	R :TTCATACACTTCACTGGCGT
APX	F :GCTTCTCTTGAGCTTTGCTGT	R :TCTGTTGGGGAACACCGAGA
SOD	F :ATGGTGCTCCTGACGATG	R :CCACTGCCCTTCCAATAAT
POD	F :GTTCTGGTTGGGCTTGG	R :TTGTCCTCGTCTGTTGGTC

F[1]: Forward primer sequence, R[2]: Reverse primer sequence.
